# Lateral entry pins and Slongo’s external fixation: which method is more ideal for older children with supracondylar humeral fractures?

**DOI:** 10.1186/s13018-022-03117-1

**Published:** 2022-04-07

**Authors:** Andreas Rehm, Joshua C. Y. Ong, Tamás Kobezda, Luke Granger

**Affiliations:** Consultant Paediatric Orthopaedic Surgeon, Cambridge University Hospitals NHS Trust, Hills Road, Cambridge, CB2 0QQ UK

We read with interest the recent publication by He and colleagues [1]. The authors reported that there was no significant difference between the groups in terms of clinical characteristics despite a huge difference in body mass index (BMI), with the K-wire group having a normal mean BMI of 22.4 and the external fixator group being obese with a mean BMI of 30.3. There was also a disparity in the sex proportions, with 30.6% girls in the K-wire and 37.5% girls in the external fixator group. Golden et al. [2] reported a negative correlation between increased BMI and range of movement (ROM) of the elbow, with an expected loss of ROM of about 11° to 17° for obese children because of a soft tissue block to full flexion and girls having a greater ROM than boys ( ~3°). He et al [1] have measured the same mean ROM for the uninjured side for both groups, instead of a reduced ROM for the obese external fixator group, which raises doubts about the reliability of their measurements with the possibility of a systematic measuring error. The authors did not describe how ROM was measured and did not conduct inter- and intra-rater reliability testing, which also raises doubts about the accuracy and reliability of their measurements.

There is a discrepancy between the mean ROM and carrying angle measurements and the Flynn scores at 6 months, with the total mean ROM and carrying angles being the same for both groups but the external fixator group having better Flynn scores for ROM and carrying angle. This suggests to us that the individual measurements for both groups were close together, with the external fixator group having a slightly wider bi-directional spread and the difference in the Flynn scores most likely not being clinically relevant to judge the outcomes between the groups.

He et al. [1] did not perform a radiographic assessment post-operatively, neither measuring Baumann’s angle, lateral capitello-humeral angle [3], relationship between anterior humeral line and capitellum [4], nor rotational alignment (lateral rotational percentage) [5].

He et al.’s [1] second additional file shows an intra-operative lateral elbow radiograph with an external fixator in place, with a loss of possibly > 50° of flexion, considering a normal lateral capitello-humeral angle of a mean of 51° [3] and the anterior humeral line not touching the capitellum. Such a severe deformity would have barely started to remodel at He et al.’s [1] 6th month follow-up, with us expecting to see a clinical flexion deficit of about 40° and hyperextension of about 30° (or more) at that point but the authors [1] did not report such findings, which also indicates to us that there is a lack of measurement accuracy. This case also contradicts the latter authors [1] claim that external fixation is a more appropriate surgical approach in older children to achieve reduction but shows instead the opposite, with external fixation having failed to achieve a good reduction. The only intra-operative lateral radiograph following reduction and external fixator fixation provided by Slongo et al [6] shows an extension deformity with possible malrotation, indicating that this is not a simple technique, which does not guarantee that a good reduction can be obtained.

He et al. [1] did not mention whether the external fixator group was provided with a cast or brace after the surgery but stated that the use of an external fixator resulted in faster functional recovery, without providing any supporting chronological evidence from the assessments at 2 and 3 months.

We noted that there was a mean delay of surgery for the external fixator group of 23.88 h. We would like to ask the authors how they explain this delay, with us wondering if it is because of only a small number of surgeons (who are not readily available) being prepared to use an external fixator for this fracture?

He et al. [1] reported a significantly shorter fracture healing time for the external fixator (4 weeks) compared to the K-wire group (5 weeks) but stated that all children had been seen at 4 weeks, which seems to be inconsistent with the mean 5-week bone healing reported for the K-wire group. Could the authors explain how they judged bone healing since we are not aware of such a bone healing assessment tool for supracondylar humerus fractures in children, with us generally using 4–5 weeks casting for children ≥ 10 years of age and 3–4 weeks for children < 10 years of age, without us having seen a case of non-union?

In conclusion, we have concerns about the accuracy and reliability of He et al.’s elbow assessments as outlined above and radiographic measurements not having been considered. The authors^1^ provided data neither support their conclusion that external fixation results in early restoration of elbow joint movement and a lower risk of joint stiffness, nor support that it is a more appropriate approach to achieve a good reduction but requires a significantly increased radiation time.

## Data Availability

Not applicable.

## Abbreviations

BMI: Body mass index
ROM: Range of movement
